# Congenital diplopodia–A rare case of duplicated lower limb: A case report

**DOI:** 10.1016/j.ijscr.2021.106390

**Published:** 2021-09-10

**Authors:** Muhammad Ihsan Kitta, Harry Supratama Azis, Novra Yuditya Santoso, Iswahyudi, Reza Romadhona Fahlevi, Ferdinand Arden

**Affiliations:** a Pediatric Consultant of Orthopaedic and Traumatology Department, Hasanuddin University, Makassar, Indonesia; b Lecturer of Medical Faculty of Muhammadiyah University, Makassar, Indonesia; c Resident of Orthopaedic and Traumatology Department, Hasanuddin University, Makassar, Indonesia

**Keywords:** Diplopodia, Supernumerary digit, Pediatric, Orthopaedic, Foot and ankle

## Abstract

**Introduction and importance:**

Diplopodia is an extremely rare case in medical history, with an even fewer cases being reported in literature. We intended to enrich the literature about diplopodia with our own case report.

**Case presentation:**

We present a case about A boy, aged one year and four months old brought by his mother to the hospital with a chief complaint of a duplicated foot in his right lower leg. Physical examination demonstrated a normal left lower extremity and a relatively well-developed duplicate foot emanating from the posterior-lateral aspect of the mid-lower right leg.

**Clinical discussion:**

Diplopodia consists of partial duplication of the foot, with or without hypoplasia or positional abnormality of the ipsilateral tibia and fibula. It must be differentiated from polydactyly where the additional structures consist of toes that may or may not have corresponding metatarsals but are devoid of tarsal bone.

Treatment should be considered case-by-case basis and tailored appropriately to suit individual needs and circumstances.

**Conclusion:**

In our case, operative treatment was done at an early walking age to provide plantigrade, functional foot. Timely surgical intervention will enable patient to adapt over time. The secondary aim is to reconstruct the foot to be more acceptable aesthetically.

## Highlights

• Duplicated lower limb is a rare case.
• Operative treatment in prewalking age would improve the patient gait.
• Treatment should be tailored to each unique case.
• Aesthetic improvement post operatively help patient confidence

## 1 Introduction

Congenital duplication of the lower extremity, either complete or incomplete, is an infrequent case. Only 26 of such cases have been reported in the literature though there have been a few reports of lower extremity duplication and associated congenital anomalies [1]. Diplopodia, or duplicated foot, is a rare congenital anomaly. It differs from polydactyly in that supernumerary metatarsal, and tarsal bones are present as well as extra digits [2], [3]. Only a few cases of this anomaly have been reported in the literature. Karchinov [2], [3] reported six cases with associated hypoplasia or aplasia of the tibial bone. Hamanishi and colleagues [4] reported a case of diplopodia with a normal tibia and fibula associated with an absent left kidney. Jones et al. [5] reported five cases of diplopodia, all with aplasia or dysplasia of the tibia. This report represents an attempt at a classification of congenital duplication of the lower extremity reported so far and a description of the treatment course in the present case.

## 2 Case report

This case report is written according to the SCARE Guidelines [6]. Boy, aged one year and four months old brought by his mother our private hospital with a duplicated foot in his right lower leg. The obstetric history is uneventful. The mother delivered at 39 weeks of gestation by spontaneous vaginal delivery. The birth weight was 3000 g. The boy was the fourth child. There was no history of teratogenic exposure during pregnancy, and there was no family history of diplopodia.

**Fig. 1:**
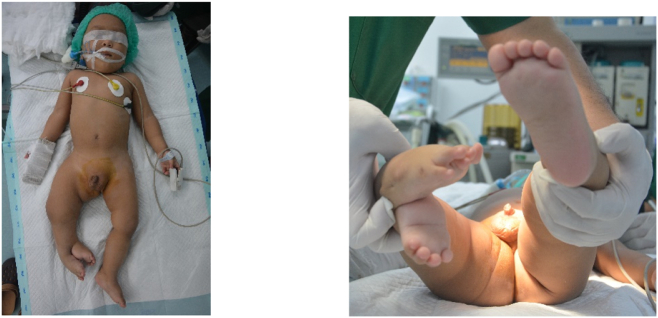
(Left) Preoperative anterior view. The additional limb originates from the lower posterior aspect of the right calf. (Right) Preoperative Inferior view. The additional limb is short, and the ankle in equinus position.

Physical examination demonstrated a normal left lower extremity and a relatively well-developed duplicate foot emanating from the posterior-lateral aspect of the mid-lower right leg (Fig. 1). The duplicate foot had four toes and appeared to have an Achilles tendon-like attachment to the leg. A strong dorsalis pedis pulse was palpated in the duplicated foot. No active motion could be seen from the duplicated foot, but movement in the native right lower extremity was normal at all joints. Pulses and sensations were normal in the native left lower extremity. No other physical abnormalities were detected. There is a 4 cm of leg length discrepancy where the right lower limb is shorter.

**Fig. 2:**
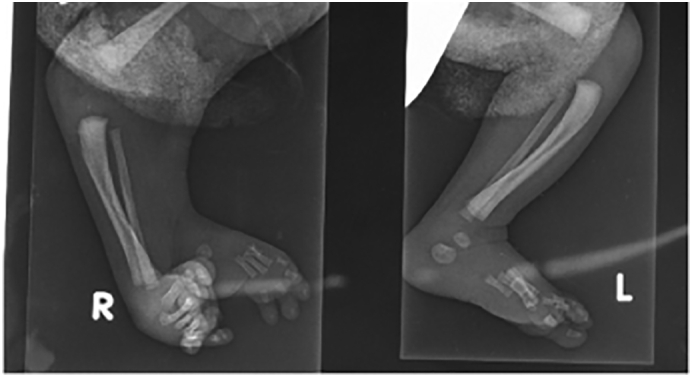
(Left) Duplicated Right foot, (Right) Normal of Left Foot.

**Fig. 3:**
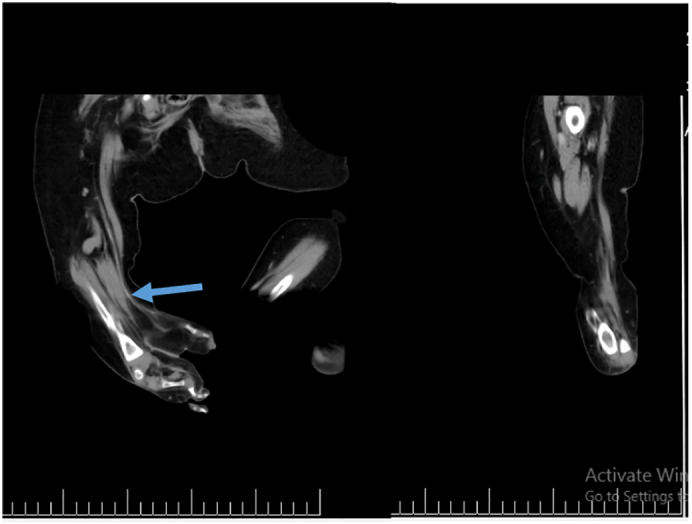
CT angiography. Blue Arrow: bifurcation of normal and duplicated artery to the duplicated foot. (For interpretation of the references to colour in this figure legend, the reader is referred to the web version of this article.)

Radiographic studies were done to evaluate both legs (Fig. 2). The duplicated foot had four toes articulating with individual metatarsals. Two ossified tarsal bones, a calcaneus, and talus, were also present. CT images (Fig. 3) revealed a near complete mirror image duplication of the right foot with its own talus and calcaneum. Both lower limbs displayed single tibia and fibula of the same size and length. The right popliteal artery was larger than the contralateral side. It was abnormal in its course and bifurcated into two main branches at the popliteal fossa. The anterior branch bifurcated at the popliteal fossa into the right anterior and posterior tibial arteries and peroneal artery. The anomalous posterior branch gave rise to two tributaries at 3 cm proximal to the native calcaneum, namely the anteromedial branch and the posterolateral branch.

**Fig. 4:**
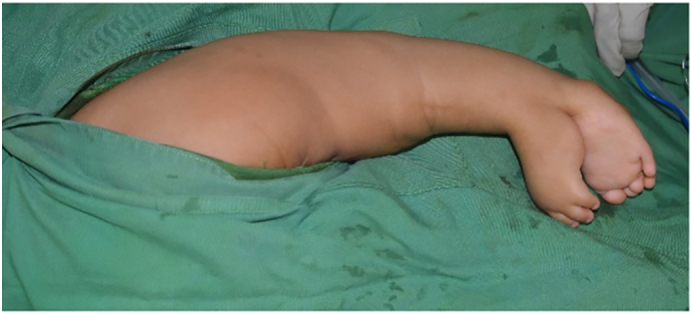
Patient positioned in prone position.

**Fig. 5:**
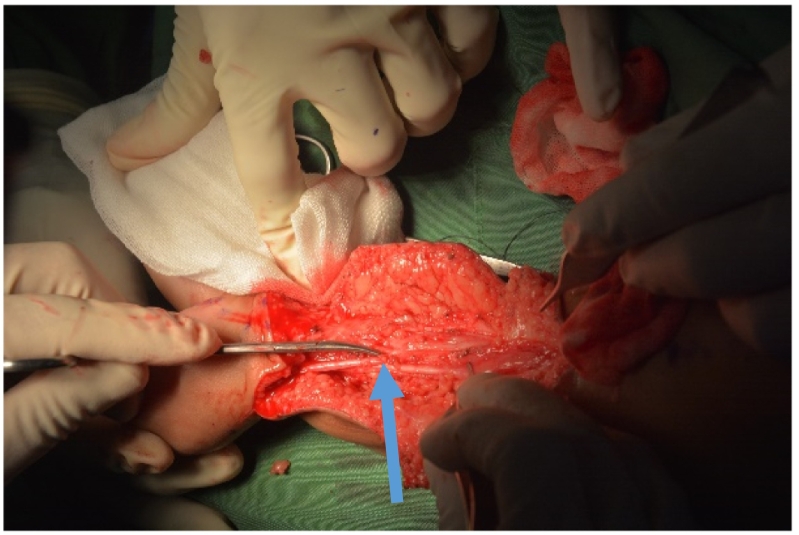
Neurovascular bundle of posterior calf. Blue arrow: bifurcation of normal and duplicated artery. (For interpretation of the references to colour in this figure legend, the reader is referred to the web version of this article.)

The operation procedure was done by our pediatric orthopaedic consultant, Dr. Muh. Ihsan Kitta, M. Kes, Sp. OT(K) whom have experience as an pediatric orthopaedic surgeon for 5 years. For surgical treatment, the patient was positioned prone on the surgical table (Fig. 4) . The zigzag surgical approach was performed. Initially, we identify the origin and insertion of the neurovascular bundle at the duplicated foot (Fig. 5), and then dissect the structure sharply.

**Fig. 6:**
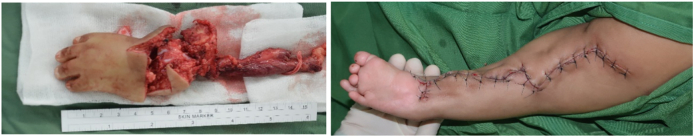
Duplicated foot was removed.

The duplicate foot was surgically removed (Fig. 6). Prior to closure, it was noted that the musculotendinous anatomy and the perfusion of the native right leg appeared otherwise normal. Dissection of the specimen revealed no muscular structures in the foot other than the aberrant gastrocnemius muscle described above.

**Fig. 7:**
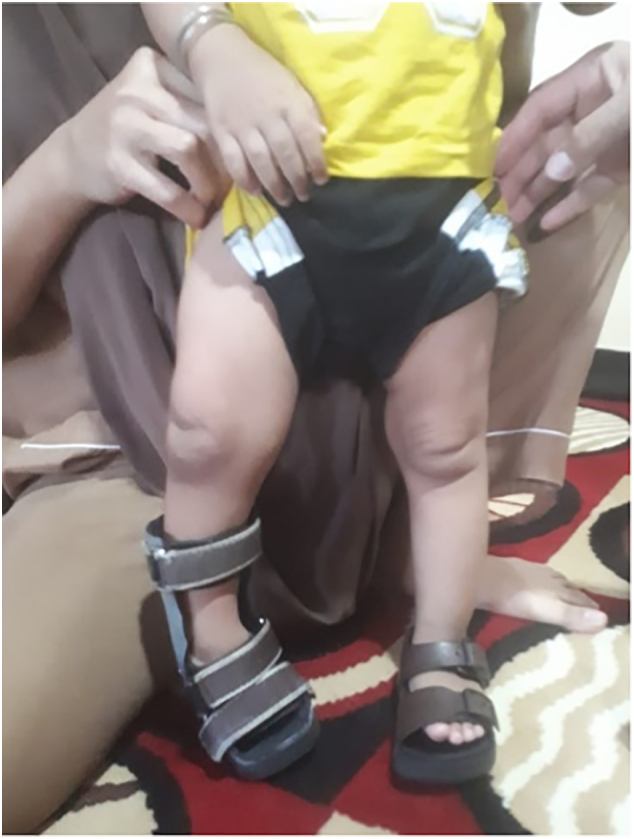
Follow up condition at 3 months of follow up.

After the surgery the patient underwent uneventful wound healing and started to walk using Ankle-Foot Orthosis without any major complaints. Fig. 7 shows the patient in 3 months of follow up.

## 3 Discussion

Diplopodia was previously described in the first publication of its kind by Karchinov [2], [7] as partial duplication of the foot with the accessory structures located on the tibial aspect. Diplopodia is less common than diplocheiria or duplication of the hand [7], [8]. In addition to supernumerary digits, duplicated foot has well-formed accessory tarsals and metatarsals, which are separated from the structures of the normal foot. It must be differentiated from polydactyly where the additional structures consist of toes that may or may not have corresponding metatarsals but devoid of tarsal bone [2], [8], [9].

Diplopodia is an extremely rare congenital deformity. Many standard textbooks do not highlight this disorder, and a small number of case reports appear to be the main source of information regarding congenital diplopodia. The condition was reported for the first time in 1966 when 4 cases were described [10], [11]. In essence, diplopodia consists of partial duplication of the foot, with or without hypoplasia or positional abnormality of the ipsilateral tibia and fibula [10], [11], [12].The condition is also known as pre-axial mirror polydactyly, or mirror foot [11], [12], and it differs from classic polydactyly, wherein supernumerary tarsals, metatarsals, and/or extra phalanges (digits) are usually present [2], [10]. As such, diplopodia often presents a monstrous appearance of the foot [2], [10], and has been associated with anal atresia and prenatal thalidomide exposure [3], [10].

Limb formation begins as limb buds from the ventrolateral body wall at the fourth week of embryonal development [7], [13]. Exposure to teratogens such as thalidomide during this period can cause deformities in extremity development [7], [13]. Until now, no apparent factors can be attributed to causing diplopodia in humans [7], [14].

Treatment for diplopodia should be tailored to suit individual needs and circumstances. The surgical intervention for our case took place in time for the patient to enjoy the privilege of a normal learning walking process and following the recommended timing in other similar reported cases [2], [7], [15], [16]. The ultimate aim of the surgery is to provide the child with a normal or near-normal functioning plantigrade foot, and also enrich the literature on various surgical techniques to deal with diplopodia cases.

Timely surgical intervention will enable the patient to adapt to the structural changes early, as the reconstructed bone will remodel over time. Furthermore, surgical intervention during prewalking phase will allow the child to focus on gait training. The secondary aim is to reconstruct the foot to be more aesthetically acceptable to the general public [7], [16].

## 4 Conclusion

Operative treatment should be done at an early walking age to provide plantigrade, functional foot. Timely surgical intervention will enable the patient to adapt over time. The secondary aim is to reconstruct the foot to be more acceptable aesthetically.

## Sources of funding

None.

## Ethical approval

The following statement applies to all listed authors:

Written informed consent was obtained from the patient's parents for publication of this case report and accompanying images. A copy of the written consent is available for review by the Editor-in-Chief of this journal on request.

## Consent

The following statement applies to all listed authors:

Written informed consent was obtained from the patient's parents for publication of this case report and accompanying images. A copy of the written consent is available for review by the Editor-in-Chief of this journal on request.

## Research registration

Not applicable.

## Guarantor

Muh. Ihsan Kitta (Pediatric Orthopaedic Consultant).

## Provenance and peer review

Not commissioned, externally peer-reviewed.

## Other relevant information

The case report complies with SCARE Guidelines [6].

## CRediT authorship contribution statement

Muh. Ihsan Kitta operated and conceived the case report.

Harry Supratama Azis analyzed the data and supervised the work.

Ferdinand Arden collected the data, contributed to the writing.

All authors discussed the results and contributed to the final manuscript.

## Declaration of competing interest

None.
